# Extracellular Vesicles: Biology and Potentials in Cancer Therapeutics

**DOI:** 10.3390/ijms22179586

**Published:** 2021-09-03

**Authors:** William C. S. Cho

**Affiliations:** Department of Clinical Oncology, Queen Elizabeth Hospital, Hong Kong, China; chocs@ha.org.hk

**Background**

Extracellular vesicles (EVs) are particles wrapped in a lipid bilayer membrane and are naturally released from cells. This kind of cargo vessel is a nanostructure, which mainly transfers lipids, proteins, various nucleic acid fragments, and metabolic components to neighboring cells or distant parts of the body through the circulatory system. EV is of great significance to the communication mechanism between cells. This Special Issue aims to collect articles to enhance our understanding of the biological characteristics of EVs and their potential applications. It features a set of high-impact articles from leading experts in the field. Through a rigorous peer-review process, a total of nineteen articles were accepted, including eight original research articles, ten review articles, and one communication article.

**Biology of EVs**

Menck et al. [1] summarized the current knowledge about the biology and composition of microvesicles (MV), as well as their role in the tumor microenvironment (TME). Increasing evidence shows that although MVs are biologically different, they have the tumor-promoting properties of endosomal-derived small exosomes in TME. Due to their larger size, they can be easily collected from the patient’s blood and characterized by conventional flow cytometry using excess molecules expressed on their surface.

Żmigrodzka et al. [2] reviewed the biogenesis and cargo molecules of platelet extracellular vesicles (PEV), as well as their effects on cancer progression. During platelet activation or apoptosis, PEV is formed, which presents a highly heterogeneous EV group and is the most abundant EV group in the circulatory system. Since the role of platelets in cancer development is well known, and PEV is the most abundant EV in the blood, its possible impact on cancer growth has been strongly discussed. The crosstalk of PEV can promote proliferation, change TME, and promote the formation of metastasis. In many cases, these functions are related to specific cargo molecules transferred from PEV to recipient cells.

Today, in multiple studies exploring TME, the interaction between tumors and macrophages is certainly interesting. Among them, tumor-associated macrophages include a subgroup that has a variety of tumor-promoting effects (including general immunosuppression) in tumor development, which can be identified based on the high expression of mannose receptor/CD206 [3].

Radiation affects not only target cells, but also neighboring cells that have not been exposed to radiation. This response is described as radiation-induced bystander effects (RIBE). Molecular communication between irradiated and unirradiated adjacent cells can trigger RIBE and out-of-field (distance) effects. Cagatay et al. [4] evaluated the changes in the spectrum of exosomes and the role of exosomes as possible molecular signaling mediators for radiation damage. After 24 hours and 15 days of irradiation, exosomes derived from the whole body or partial body of irradiated mouse organs were transferred to recipient mouse embryonic fibroblast (MEF) cells. The changes in cell viability, DNA damage and calcium, reactive oxygen species, and nitric oxide signals were compared with exosome-treated MEF cells from unirradiated mice. Their results showed that whole and local irradiation would increase the number of exosomes and induce changes in MEF cells treated by exosomes.

**EV Isolation and Characterization**

Many isolation techniques typically discard large EVs in the early stage of sEV or exosome isolation protocols. Johnson et al. [5] described the stepwise separation and characterization of large EV subsets in a medulloblastoma cell line using fluorescent light microscopy, transmission electron microscopy, and tunable resistance pulse sensing. They developed a labeling and strict gating strategy to explore the expression of EV markers (CD63, CD9, and LAMP 1) on a single EV in a wide heterogeneous population. Their data strongly support the exploration of large EVs in clinical samples to obtain potential biomarkers, which are very useful in diagnostic screening and disease monitoring.

EV concentrations often result in low final yields or severe contamination of vesicle samples, which greatly limits further applications and data reproducibility, and contamination greatly affects a wide range of functional studies. Simon et al. [6] described a new combination of three well-known methods (size exclusion chromatography (SEC), Western blotting, and transmission electron microscopy) to obtain medium to high yields of EVs while reducing protein contamination. They believe that this method may have great benefits for in vitro and in vivo functional studies.

Efforts have also been made to standardize the separation and characterization techniques of sEV. Current protocols often result in the co-separation of soluble proteins or lipid complexes with other EVs. Bordas et al. [7] reported an optimized protocol for the isolation of sEV from human and murine lymphoid tissues. To separate sEV from freshly resected human lymph nodes and mouse spleen, two different methods were compared: (1) ultracentrifugation on a sucrose density pad and (2) a combination of ultracentrifugation and SEC. The purity of sEV preparations was analyzed using Western blotting, nanoparticle tracking analysis, and electron microscopy. Their results clearly demonstrated the superiority of SEC in improving the yield and purity of sEV. 

On the other hand, it is questionable whether clinical laboratories can conduct in-depth research through flow cytometry to evaluate EV surface cargo in various diseases. Lucchetti et al. [8] reported the difficulty of evaluating small and medium-sized EVs through traditional flow cytometry. Running a sample of medium EVs stained with equal amounts of Calcein-green and Calcein-violet, they found that the cluster detection produced false double positive events. This phenomenon was significantly reduced by sample dilution, but it was not completely eliminated. In addition, running highly diluted samples required a longer cytometer time. Their findings question the routine applicability of traditional flow cytometry in EV analysis.

**Therapeutic Potential of EVs**

It is well known that the potential use of EVs as therapeutic agents lies not only in their cell membrane-bound components but also in their cargo. Jurj et al. [9] highlighted the characteristics of EV involvement in cancer cells, paying special attention to those molecular processes that were affected by EV cargo. In addition, they explored the role of RNA types and proteins carried by EVs in triggering the drug resistance phenotype. Interestingly, in various in vivo and in vitro studies and multiple clinical trials, engineered EVs have been proposed as therapeutic agents.

The composition of exosomes and the possibility of interacting with cells make exosomes a multifaceted regulator of cancer development. Lorenc et al. [10] discussed the role of tumor-derived exosome (TEX) in the progression of prostate cancer and the potential use of exosomes in the management of prostate cancer. The biophysical properties of EVs (such as stability, biocompatibility, permeability, low toxicity, and low immunogenicity) are the key to the successful development of innovative drug delivery systems. EV has enhanced circulation stability and biological barrier permeability, so it can be used as an effective chemotherapy carrier to improve the regulation of target tissues and organs. Exosomes can deliver different types of cargo and target specific cells. They may help deliver chemotherapy drugs, natural products, and RNA-based cancer gene therapy [11].

The combination of ultrasound and microbubbles has been shown to trigger cancer cells to release EVs. Yuana et al. [12] used microbubbles-assisted ultrasound (USMB) to load the model drug CellTracker green fluorescent dye (CTG) or bovine serum albumin coupled with fluorescein isothiocyanate (BSA FITC) into primary human endothelial cells in vitro. They found that USMB loaded CTG and BSA FITC into human endothelial cells and triggered the release of EVs containing these compounds in the cell supernatant within two hours after treatment. The amount of EV released seems to be related to the increase in ultrasound acoustic pressure. They concluded that USMB can load model drugs into endothelial cells and trigger the release of EV-carrying model drugs, highlighting the potential of EV as a drug nanocarrier for future cancer drug delivery.

Sun et al. [13] elaborated about the role of EV in the development of cancer therapeutics. They emphasized that some EVs (such as tumor exosomes) have a tumor-homing tendency, which has led to the use of EV as a drug carrier to effectively provide cancer treatment. The results of preclinical research and early clinical trials were mainly reviewed. For example, a phase I clinical trial designed to evaluate the ability of plant sEVs to prevent oral mucositis during chemotherapy and radiotherapy of head and neck cancer revealed the potential of using plant sEVs to reduce side effects during cancer treatment. It was also reported that isolating fibroblast-like mesenchymal cell-derived sEV and loading siRNA or shRNA targeting *KRAS* mutation (*KrasG12D*) could more effectively inhibit the progression of pancreatic ductal adenocarcinoma in vitro and in vivo compared with other drug carriers.

Interestingly, human breast milk (HBM) is an irreplaceable source of nutrition for early infant growth and development. Kim et al. [14] reviewed the various components of HBM (especially exosomes and miRNA) and their therapeutic potential for cancer. Milk-derived exosomes play a variety of physiological and therapeutic functions in cell proliferation, inflammation, and immune regulation, mainly due to their cargo molecules (such as proteins and miRNA). Exosomal miRNA is not affected by enzymatic digestion and acidic conditions, and it plays a key role in immune regulation and cancer. In addition, milk-derived exosomes have been developed as drug carriers for the delivery of small molecules and siRNA to tumor sites.

Although cancer treatments encounter physiological obstacles in TME, they must be delivered to their target to improve efficacy and reduce toxicity. Choi et al. [15] summarized the biological function and therapeutic potential of exosomes as diagnostic biomarkers and drug delivery vehicles for cancer treatment. They also explored whether exosomes could be used as effective nanocommunicators to promote drug design for personalized cancer immunotherapy.

Despite the tremendous technological advances in the treatment of head and neck squamous cell carcinoma (HNSCC), the overall survival rate is usually low. Ebnoether and Muller [16] summarized the diagnostic and therapeutic applications of exosomes in HNSCC. They also reviewed the impressive preliminary results obtained in recent studies.

Since TEX is involved in the crosstalk between cancer and immune cells, it plays a key role in suppressing the anti-tumor immune response to promote tumor progression. Most of the available information about the molecular composition and function of TEX was obtained using small EV (sEV) isolated from the supernatant of cancer cell lines. However, new data linking plasma TEX levels to cancer progression focuses on TEX in the peripheral circulation of patients as a potential biomarker for cancer diagnosis, development, activity, and response to treatment. Zebrowska et al. [17] published an article on the signaling of melanoma cell-derived sEV, with particular emphasis on exosome-mediated signal transduction between melanoma cells and the host immune system. They argue that the signaling of TEX may impact melanoma progression.

Dobra et al. [18] initiated the first proteomic study comparing whole-serum and serum-derived sEV. They aimed to determine the characteristic protein fingerprints associated with central nervous system (CNS) tumors. A total of 96 human serum samples were obtained from four patient groups (glioblastoma multiforme, brain metastases from non-small-cell lung cancer, meningioma, and lumbar disc herniation). Among the 311 identified proteins, 10 whole serum proteins and 17 serum-derived sEV proteins showed the highest difference between each group. A total of 65 proteins were significantly enriched in serum-derived sEV samples, while 129 proteins were significantly reduced compared with whole-serum sEV samples. Based on principal component analysis, serum-derived sEV was more suitable for distinguishing patient groups and has a greater potential for monitoring CNS tumors than whole-serum sEV.

Glioblastoma is a devastating disease, and there is an urgent need for biomarkers that can predict prognosis. Tzaridis et al. [19] analyzed a set of serum microRNAs (miRNAs) in the EV from glioblastoma patients and examined their relevance to the prognosis of these patients. Using RT-qPCR and CD44 immunoprecipitation (sinusoidal endothelial cell + CD44), the levels of 15 miRNAs in EVs separated by SEC were compared with those of glioblastoma patients and healthy volunteers. Combining miR-15b-3p in serum or miR-106a-5p in CD44 immunoprecipitation EVs with any of the other three miRNAs in CD44 immunoprecipitation EV could stratify the prognosis of glioblastoma patients.

Transporting the biologically active cargo of MVs to target cells can affect their fate and behavior and change their microenvironment. Szyposzynska et al. [20] evaluated the biological activity of MV derived from human immortalized mesenchymal stem cells of adipose tissue-origin (HATMSC2-MVs) against two ovarian cancer cell lines ES-2 and OAW-42. They prove that HATMSC2-MVs can effectively metastasize to ovarian cancer cells and inhibit cell proliferation, apoptosis, and/or necrosis through different pathways, which may be related to the presence of different anti-tumor factors secreted by ES-2 and OAW-42 cells.

**Conclusions**

We hope that all these efforts will improve our understanding of the EV world and provide some insights into its potential clinical applications. With its multiple encapsulated macromolecules and powerful extraction platform, we envision a more comprehensive understanding of EV biology through multi-omics approaches.
